# Uncertainty and Nursing Needs of Parents with Pediatric Cancer Patients in Different Treatment Phases: A Cross-Sectional Study

**DOI:** 10.3390/ijerph18084253

**Published:** 2021-04-16

**Authors:** Mijeong Park, Eunyoung E. Suh, Soo-Young Yu

**Affiliations:** 1 Department of Nursing, Seoul National University Children’s Hospital, Seoul 03080, Korea; mijeong@snuh.org; 2 Center for Human-Caring Nurse Leaders for the Future by Brain Korea 21 (BK 21) Four Project, Research Institute of Nursing Science, College of Nursing, Seoul National University, Seoul 03080, Korea; esuh@snu.ac.kr; 3 College of Nursing, Seoul National University, Seoul 03080, Korea

**Keywords:** uncertainty, pediatrics, parents, family nursing, oncology nursing

## Abstract

The survival rate of pediatric cancer has increased to 80%, but long-term treatment is required. During treatment, parents experience uncertainty, which affects parents’ quality of life and, even worse, their children’s health; however, the variation of that uncertainty remains under-studied. Thus, it is crucial to understand parents’ nursing needs in each distinct treatment phase to develop relevant educational content. This study investigated the uncertainty level and nursing needs of parents according to their children’s treatment phase. This cross-sectional comparative descriptive study collected survey data from 119 people at a tertiary hospital from December 2017 to April 2018. Nursing needs were ascertained using open-ended questions, and data were analyzed using quantitative content analysis. The uncertainty levels of parents of pediatric cancer patients showed statistically significant differences across treatment phases (F = 8.209, *p* < 0.001). Parents’ uncertainty was higher in the treatment initiation phase (87.77 ± 13.43) and when treatment was ongoing (83.33 ± 15.10) than in the post-treatment phase (75.35 ± 12.82). All three groups had nursing needs regarding infection control, diet, daily activities of living, and prognosis. Parents’ uncertainty levels and nursing needs differed across treatment phases, suggesting a need for tailored education programs to provide practical support to parents of pediatric cancer patients in each phase.

## 1. Introduction

Malignant tumors in childhood are one of the most common causes of death in pediatric diseases. The incidence rate of childhood cancer in South Korea is 14.6 per hundred thousand children below 15 years, and the incidence rate is also increasing [1,2]. Although the current 5-year survival rate of pediatric cancer has improved to more than 80% in Korea, a cancer diagnosis in children is a lifetime event that can cause a crisis within the family. Moreover, the physical and psychological pain that children and their families experience does not necessarily decrease over time [3,4].

When a child is diagnosed with cancer, the caregiver of the child experiences negative emotional responses such as guilt, denial, anger, and helplessness. Parents often spend 24 h per day with their children in the hospital, immersing themselves in their child’s care. A childhood cancer diagnosis is one of the most painful experiences that parents can face, and caregivers of pediatric cancer patients experience extreme stress and uncertainty [3]. This uncertainty could affect the prognosis of pediatric cancer patients. Therefore, nursing care that considers parents as well as children has benefits for children’s health and safety.

Parents experience various forms of stress and uncertainty, including fear of future recurrence, throughout the disease process. First, when a child is diagnosed with cancer, parents plan a daily routine that prioritizes coping with the child’s disease. While determining the treatment method, parents play multiple roles, such as providing emotional support and financial support to their child as a patient’s guardian [5]. As the patient begins to receive cancer treatment in earnest, parents face new events such as repeated diagnostic tests, drug injections, invasive procedures, and physical changes in their child. After months of complicated treatment, which may include chemotherapy, radiation therapy, surgery, and transplantation, parents of children with cancer experience social difficulties as well as physical exhaustion and financial burdens [6]. Even after the patient’s treatment has ended, changes in health conditions occur, and adaptation to a new lifestyle is required. There can also be anxiety about the occurrence of later side effects or recurrence [7]. Parents accept a “life-lasting emergency” as the new normal, from cancer diagnosis to the entire process of treatment [8]. Their uncertainty can also include concern over errors in the treatment process, suspicion of treatment failures, and stress in response to the transition to normal life.

Uncertainty is considered to be a state where it is difficult to accurately determine the meaning of the situation as it pertains to the disease in the course of an individual’s disease experience [9]. It is necessary to provide disease-related information and explain treatment steps to parents, who function as primary care providers for children with pediatric cancer [9].

In particular, the level of uncertainty can change and be reevaluated over time in patients with chronic diseases such as cancer [10], and Cohen (1995) [11] pointed out that uncertainty levels vary with the severity of the disease, such as during the acute versus diagnostic stages. A relatively high level of uncertainty remained even after all treatments had ended in a study that measured the level of uncertainty by treatment phase [12]. Uncertainty persists not only in the early stages of the cancer diagnosis and during treatment but even after treatment is over. Furthermore, uncertainty can cause depression, a decrease in quality of life, and post-traumatic stress disorder [13]. These studies suggest the need for a nursing strategy that reduces uncertainty even after treatment is completed.

Parents’ coping strategies may not be comprehensive if they do not manage changes in uncertainty levels at each treatment phase without structured support [14]. Improving families’ ability to cope with uncertainty in disease situations will be beneficial for long-term disease management. Care during each disease stage is necessary for those affected by pediatric cancer because patients are repeatedly hospitalized and discharged during their treatment period, and care at home is critical even after discharge from the hospital [15]. A qualitative study exploring the parenting experiences of parents whose child had cancer in South Korea reported that parents experienced confusion as their child adapted to school [16].

The nursing needs of children diagnosed with cancer may differ depending on the treatment phase as they continue to grow physically and mentally during the treatment process. Various needs exist from diagnosis to the termination of treatment. It is crucial to know what is necessary to care for the child’s unfamiliar symptoms related to the disease as children have difficulty expressing their discomfort or identifying new symptoms on their own.

Parents’ awareness of information regarding their child’s disease affects their uncertainty level. Education for parents of pediatric cancer patients is mainly conducted at the diagnosis stage, and serial education according to the treatment phase is not typically provided in clinical practice. In order to provide appropriate education according to the treatment phase, it is necessary to identify which nursing needs are present at the time of chemotherapy or the other treatment. However, most studies have examined the anxiety, uncertainty, and nursing needs of parents of pediatric cancer patients during particular phases of the treatment process [6,7,8], instead of exploring the full scope of the period from diagnosis to the end treatment.

This study aimed to investigate the uncertainty and nursing needs of parents of children diagnosed with pediatric cancer in three phases: (1) treatment initiation, (2) ongoing treatment (consolidation therapy, maintenance therapy, etc.), and (3) post-treatment. The goals of this study were (a) to investigate the degree of uncertainty parents face according to each treatment phase of pediatric cancer, (b) to explore qualitative differences in the uncertainty faced by parents of pediatric cancer patients according to treatment phase in four subdomains (ambiguity, lack of clarity, lack of information, and unpredictability), and (c) to identify parents’ nursing needs for each treatment phase of pediatric cancer.

## 2. Materials and Methods

### 2.1. Study Design, Setting and Samples

This was a cross-sectional comparative descriptive study that identified parents’ uncertainty levels and nursing needs in each treatment phase of pediatric cancer. The criteria for participation in this study were parents with a child ongoing chemotherapy for pediatric cancer or whose treatment for pediatric cancer had recently ended. This study’s subjects were the guardians of children admitted to a pediatric hematology-oncology ward and who visited outpatient clinics at S University Hospital in Seoul, South Korea. According to the international classification, childhood cancer included both blood cancer and solid tumor diagnoses [17]. The sample size was calculated using G*Power version 3.1.9.2, with the significance level (α) = 0.05, power = 0.80, and the effect size (F^2^) = 0.3. The minimum number of subjects required for one-way analysis of variance (ANOVA) was 111 [18]. A total of 120 parents of patients were selected with an expected attrition rate of 10%. Patients were divided into groups by treatment phase: treatment initiation, ongoing treatment, and post-treatment. One person dropped out during the study. A total of 119 people participated as the final subjects, including 39 participants in the treatment initiation group, 40 in the ongoing treatment group, and 40 in the post-treatment group.

### 2.2. Measurements

#### 2.2.1. Data Collection/Procedure

Data collection was conducted for about 5 months, from December 2017 to April 2018. Recruitment of participants in this study was conducted after setting the inclusion criteria for each group according to the three treatment phases, including ‘treatment initiation phase’, ‘on-going treatment phase’, and ‘post-treatment phase’. Then this study was introduced to patients and parents who met the criteria within a certain period: in working hours of the outpatient department for five months. The researcher explained the purpose and content of the study to the parents of pediatric cancer patients who were experiencing ongoing treatment or had visited the outpatient cancer clinic at S University Hospital in Seoul, South Korea. When a parent agreed to participate in the study, they were given a structured questionnaire that was self-administrated.

Data were collected in the patient’s room or education room for parents of pediatric cancer patients at the time of diagnosis. Parents of the patients who were hospitalized for the first time in the pediatric hematology-oncology unit were recruited in the ‘treatment initiation phase’. Parents whose children were receiving at least two cycles of chemotherapy to treat a solid tumor, consolidation chemotherapy, or maintenance therapy before hematopoietic stem cell transplantation for a hematologic malignancy were included in the ‘ongoing treatment phase’ group. Parents of pediatric cancer patients in the ‘post-treatment group’ were recruited after chemotherapy ended at visits to the outpatient clinic at the children’s hospital. The survey took about 15 min to complete. A total of 120 questionnaires were distributed 40 per each phase group, and 119 were ultimately analyzed after one person dropped out from the ‘treatment initiation phase’ group.

#### 2.2.2. Instruments

- Uncertainty level

The Parent’s Perception of Uncertainty Scale-Family Member (PPUS-FM) developed by Mishel (1983) [14] was translated into Korean by Oh and Kim (2015) [19]. The PPUS-FM consists of four subdomains, which measure four dimensions of uncertainty, with 13 questions on ambiguity, nine questions on lack of clarity, five questions on lack of information, and four questions on unpredictability, comprising a total of 31 questions. Questions were answered using a 5-point Likert scale, with 1 point meaning “not at all” and 5 points meaning “very much”. The lowest possible total score is 31 points, and the highest possible total score is 155 points. A higher score indicates a higher level of uncertainty. Cronbach’s alpha was 0.81 when the instrument was developed [14] and 0.84 in the study presenting the South Korean translation of the instrument [19]. Cronbach’s alpha was 0.88 in this study.

- Nursing needs

Open-ended questions were developed by the researcher and asked after the survey to identify the nursing needs of parents of pediatric cancer patients. These open-ended questions, answered in a self-administrated format, were as follows: (1) What information would you most like to know about your child’s illness and treatment? (2) What do you need help with during treatment? What makes you think so? (3) What kind of support is most required while taking care of your child?

### 2.3. Data Analysis

The data collected using the survey were statistically analyzed and processed using SPSS for Windows version 22.0 (IBM Corp., Armonk, NY, USA). Demographic characteristics were calculated using frequency, percentage, mean, and standard deviation. The difference in the mean score of uncertainty by treatment phase and the difference in the subdomain was analyzed using one-way ANOVA, and the Scheffé test was used to perform post hoc testing of significant variables. Answers to open-ended questions about nursing needs according to treatment phase were analyzed using quantitative content analysis [20]. In the process of inductively analyzing qualitative data, a temporary theme/category was first derived, and then the coding scheme was refined. This analysis process entailed the process of deriving the final theme through peer review by two authors.

## 3. Results

### 3.1. General Characteristics of Parents and Their Children with Pediatric Cancer

#### 3.1.1. General Characteristics of Pediatric Cancer Patients’ Parents

Table 1 shows the general characteristics of a total of 119 respondents. Most of the parents of childhood cancer patients were women (*n* = 105; 88.2%), and the median age was 39 years, with a range of 23–55 years. Sixty-one (51.3%) parents identified as religious, and 58 (48.7%) did not. Ninety-four people (79.0%) had a college degree or higher, and 35 people (29.4%) were employed. Monthly household income exceeded USD 4000 (or KRW 4,000,000) for 50 people (42.0%).

#### 3.1.2. General Characteristics of Children with Childhood Cancer

Table 2 shows the general and disease-related characteristics of pediatric cancer patients. There were 69 males (58.0%) and 50 females (42.0%). The most common type of cancer was leukemia in 47 patients (39.5%), followed by brain tumors in 16 patients (13.4%), non-Hodgkin lymphoma in 14 patients (11.8%), neuroblastoma in 8 patients (6.7%), and osteosarcoma in 8 patients (6.7%). The median age of the pediatric cancer patients was 3 years, the lowest age was 0 years, and the highest age was 18 years. The largest age group comprised patients over 11 years old, with 43 patients (36.1%), followed by the group consisting of 36 patients (30.3%) between 1 and 5 years old. The time since the end of cancer treatment was less than 12 months for 25 out of 40 children in ‘post-treatment phase’ group. The rest was 1–4 years after cancer treatment.

### 3.2. Parents’ Uncertainty Level by the Treatment Phase of Pediatric Cancer Patients 

Table 3 shows the uncertainty score according to treatment phases. The parents’ uncertainty level was 87.77 ± 13.43 in the treatment initiation group, 83.33 ± 15.10 in the ongoing treatment group, and 75.35 ± 12.82 in the post-treatment group. The uncertainty level was significantly different according to the treatment phase (F = 8.209, *p* < 0.001).

The difference in mean scores for each subdomain of uncertainty was tested with one-way ANOVA. Additionally, the mean difference between the three groups was further verified statistically using the post-hoc test: Scheffé. The mean score decreased in all four domains for the post-treatment group. The decreases in ambiguity, lack of clarity, lack of information, and unpredictability were all statistically significant. The mean score of ambiguity was statistically significantly higher in the treatment initiation group and ongoing treatment group than in the post-treatment group (F = 7.49, *p* = 0.001). The mean score for lack of clarity was statistically significantly higher in the treatment initiation group than in the post-treatment group (F = 3.41, *p* = 0.036). Lack of information was significantly higher in the treatment initiation group than in the ongoing treatment and post-treatment groups (F = 5.06, *p* = 0.008). The average unpredictability score was statistically significantly higher in the treatment initiation group and the ongoing treatment group than in the post-treatment group (F = 8.20, *p* < 0.001).

**Figure 1:**
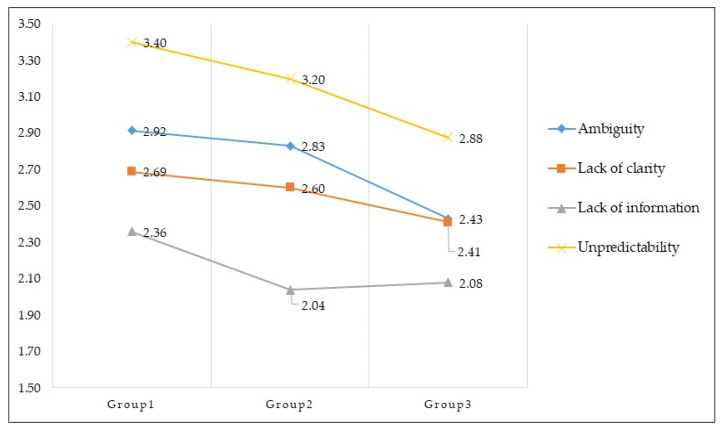
Uncertainty comparison according to treatment phase across four subdomains. Group 1 = treatment initiation phase; Group 2 = ongoing treatment phase; Group 3 = post-treatment phase.

Figure 1 shows the uncertainty level according to the treatment phase across four subdomains as a graph. The x-axis is the group according to the treatment phase, and the Y-axis is the uncertainty value, indicating the position of the average value on a 5-point scale per item. In particular, parental uncertainty was significantly reduced at the end of treatment compared to the average score of parental uncertainty at diagnosis and ongoing treatment.

Table 4 shows the uncertainty levels of scores for each item measured by the PPKS-FM scale across the treatment phase. The questions with high average scores for uncertainty were different between treatment phases. First, the questions with a high average score in the treatment initiation group were “I have a lot of questions without answers” (4.21 ± 1.00) and “It is unclear how bad my child’s pain will be” (3.72 ± 1.19). The questions with a high average score within the ongoing treatment group were “Because of the treatment, what my child can and cannot do keeps changing” (3.88 ± 0.72), “I have a lot of questions without answers” (3.85 ± 0.83), and “It is unclear how bad my child’s pain will be” (3.63 ± 1.03). Among the post-treatment group, the questions with high uncertainty scores were “I have a lot of questions without answers” (3.73 ± 0.96), “Because of the treatment, what my child can and cannot do keeps changing” (3.68 ± 1.02), and “I can predict how long the patient will be sick” (3.40 ± 1.01). The items with a high score in all three groups were “I have a lot of questions without answers” and “Because of the treatment, what my child can and cannot do keeps changing”.

### 3.3. The Nursing Needs of Parents According to the Treatment Phase of Pediatric Cancer Patients

The nursing needs of parents of pediatric cancer patients were determined by asking open-ended questions developed by the researcher. The self-administered qualitative data were analyzed using the quantitative content analysis method. The parents were asked to answer three open-ended questions pertaining to what information they needed most, the aspects of care they needed the most help with, and the care they needed themselves. The data obtained from parents was analyzed without distinguishing between the three questions, and the respondents’ answers were considered in their entirety. The most frequently used words, illustrated expressions, semantic content, and implicit content were classified as themes.

Table 5 describes the themes by parents according to the treatment phase. Parents described a total of 490 opinions. Those who were in the ongoing treatment group shared the most opinions. The most frequently mentioned needs across all respondents in each phase were good diet management during the treatment period, proper environmental management for infection control, awareness of the child’s acceptable activity level, a clear explanation of their child’s current health status, and explanations of the disease prognosis (Table 5). The number in parentheses next to the theme indicates the frequency with which this theme appeared among the responses, with a high number indicating a heightened need among respondents.

#### 3.3.1. Nursing Needs in the Treatment Initiation Group

Parents of pediatric cancer patients did not initially understand everything when first being told about their child’s cancer diagnosis. They vaguely wondered whether knowledge obtained through various channels was correct and wanted information to be verified by medical staff. Respondents wanted to know the odds of their child being cured, whether their child could be completely cured, and the disease’s prognosis based on the treatment method they choose.

*“I wonder if my child can live the same life as a normal person after a complete recovery. [six times].”*

*“What I want to know the most is the probability of complete recovery after medical treatments. Psychological treatment will also be necessary. [23 times].”*

*“I am curious about life after treatment for people who had the same type of disease as similar age of my child. I want to know how they are adjusting to family and social life and at what rate. [nine times].”*

When comparing the nursing needs of parents of pediatric cancer patients by treatment period, parents from the treatment initiation group most commonly needed information regarding the treatment success rate and the post-treatment prognosis. Since respondents in this group had not yet started or were still deciding whether to start chemotherapy, they worried how their children would respond to treatment and if they could fully recover. They needed to know whether they could return to normal life before the treatment was over. Despite having access to extensive information through the internet and acquaintances, this concern was widespread. More specific nursing needs, such as acclimation to unfamiliar therapeutic agents or side effects, typically arose when treatment began.

#### 3.3.2. Nursing Needs in the Ongoing Treatment Group

As their children received chemotherapy, parents wanted more detailed information about medication used for specific treatment and its effect on their child’s progress. They also wondered how to cope with psychological problems resulting from treatment and when chemotherapy side effects would end.

*“I am curious why the disease occurred and whether it will recur after the end of treatment. Also, will there be any complications or other factors which could be harmful to the human body due to side effects during chemotherapy? Frequent detailed explanations of the patient’s current status during treatment are required. The child is being treated based on unfounded beliefs whereas the information or physical status of the child’s treatment is unknown. [10 times].”*

*“When introducing a new treatment or drug, I would like an exact explanation of why it is used, its side effects, its intended effects, test results with detailed descriptions, and the exact treatment process. [14 times].”*

Parents of patients in the ongoing treatment group had specific questions regarding practical knowledge for caring for pediatric cancer patients as the treatment proceeded and they experienced symptoms, rather than concerns about their lack of a full understanding at the treatment initiation phase. Participants demonstrated psychological and emotional nursing care needs related to the difficulties pediatric cancer patients faced due to changes in daily living activities, changed appearance, and relationship with friends.

#### 3.3.3. Nursing Needs in the Post-Treatment Group

At the end of treatment, parents needed information about the disease prognosis, adjustment to daily life, and their child’s return to school or society. They also wanted to know how to manage long-term side effects and sequelae that may appear after treatment.

*“I heard that returning to normal life is possible, but I am curious as to what is allowed and what is not. I am also concerned about the risk of infection. Thus I’m cautious about my outside activities as a parent. Also, I wonder if my child’s immune system is weaker than other person’s after treatment is finished. With my child no longer seeing the doctor as often as they did during treatment, I feel anxious when my child is in poor condition. [27 times].”*

Some parents answered that regular education is necessary regarding long-term management, such as side effects and sequelae prevention. Parents stated that they wanted to fully understand the whole process by receiving regular explanations of the things that at first seemed incomprehensible at the beginning of the treatment stage. As the explanations parents received were unfamiliar and challenging to grasp when their children were first diagnosed with cancer, parents wanted consistent communication without confusion through systematic education from nursing professionals.

*“It’s not easy [for my child] to move anymore because of complication: knee necrosis. I would like to know how to alleviate some of the complications and side effects during the treatment process. It will be more helpful to participate in complication relief program information depends on the treatment process in advance. [eight times].”*

Some parents commented on the need for social adaptation training and psychological support for their child to return to school and the community after long-term hospitalization. Parents wanted to know how to get information through the internet without having to visit the hospital.

*“I would appreciate if I could receive detailed explanations or things to keep in mind through lectures or social media. I am afraid of my child joining kindergarten, school, and community life even after treatment. [seven times].”*

These nursing needs seemed to reflect parents’ fears of recurrence and difficulties for their child as they adapt to society and return to daily life after treatment is finished. After treatment, parents’ access to information is limited due to spending less time in the hospital, which indicates a significant need for more information regarding their children’s care.

## 4. Discussion

This study is the first attempt to measure the uncertainty level of parents of pediatric cancer patients according to the treatment phase. The results of the investigation, which used the PPUS-FM developed by Mishel, showed that uncertainty levels were significantly higher in the parents of pediatric cancer patients in the treatment initiation and ongoing treatment groups than in the post-treatment group.

The uncertainty levels observed in this investigation were markedly lower than those of another study that surveyed fathers of pediatric cancer patients whose children were undergoing chemotherapy in China [21]. A possible explanation for this might be gender difference between parents since most of the parents included in our study were women.

The parents who participated in our study were also generally young and were more likely to be able to obtain information through various media or the internet, which could have affected their level of uncertainty.

The current study found that unpredictability was the subdomain of uncertainty with the highest score among parents of pediatric cancer patients. Ambiguity was the second-highest subdomain. When a child is hospitalized due to illness, parents can experience a high level of uncertainty and ambiguity due to not knowing the cause of their child’s condition and being unable to predict what treatments will be required. This study supports evidence from a previous study of mothers of hospitalized children in South Korea [22]. However, these findings contradict those of an earlier study, which found that lack of clarity was the subdomain with the highest score among mothers of pediatric cancer patients [23], and another study that found the highest average score for the “lack of information” subdomain in adult cancer patients [24]. Uncertainty may differ depending on survey respondents’ disease characteristics or demographic characteristics, and these results should be interpreted with some caution.

Disease-related uncertainty is a result of psychological and physical health conditions and affects the symptoms of mothers with chronic diseases [12]. The average uncertainty score was highest at the time of diagnosis, but uncertainty levels can be reduced by providing necessary information at the initiation of treatment. However, providing too many educational materials in a short time could cause care to be burdensome. It is also known that sudden and intensive demands can cause anxiety or depression and negatively affect one’s well-being [11]. This finding suggests the importance of providing education that considers not just the information being shared but also the time at which it is shared to reduce uncertainty.

One unanticipated finding was that the uncertainty level of respondents in the post-treatment group was lower than that of respondents in the treatment initiation group, which is the opposite result from that of a study in Sweden that showed a high uncertainty level at the end of treatment [12]. This somewhat contradictory result may be because the uncertainty level was measured not at the end of the treatment but rather slightly after the end of treatment. Thus, caution must be applied in interpreting these findings.

The commonly reported needs of the parents of pediatric cancer patients in all treatment phases were diet education, environmental infection control, and provision of information about the current patient’s status and prognosis. The reason for the high need for diet education and environmental infection control throughout the entire treatment phase may be that these needs are within a parent’s control and are relevant to their own and their children’s daily activities.

Parents of pediatric cancer patients wanted to know about the patients’ current health status and prognosis, as well as the possibility of recurrence. These results are in line with previous studies investigating the nursing needs of families of children with cancer in South Korea and Jordan. The education and information sections showed the most demand from South Korean family members [25]. Parents of pediatric cancer patients in Jordan demanded high levels of information on testing and treatment, but these needs were often unfulfilled [26].

Our findings are consistent with studies of parents of hospitalized children in South Korea. Counseling and education were ranked second to direct nursing in a previous study [27]. This finding was also reported in a survey with family members of pediatric patients, which demonstrated the need for nursing care to identify health conditions, explain the reasons and results of medicine administration, and respond to parents’ questions in good faith [28,29].

This combination of findings suggests that medical staff should offer regular explanations, guidance, and sincere answers during therapeutic procedures. Parents’ accurate understanding of their child’s situation is critical in their child’s treatment and care [24]. When parents receive a complete explanation, it gives parents an opportunity to effectively support and reassure their child in an unfamiliar environment [30]. Considering parents’ uncertainty levels, counseling and education should be more prevalent in child care practice in order to accommodate parents’ nursing needs.

The study also found a high demand for explanations of the physical symptoms resulting from the treatment process and the timeline for treatment up to its conclusion. These results suggest that specific nursing needs related to symptom resolution are linked to known medications that the child receives while undergoing treatment. Parents wanted to know when there were changes in the treatment plan during the actual treatment period compared to the plan established at the start of treatment. This was not always resolved since most parents only saw medical staff briefly unless problems requiring intensive care due to complications occurred.

In addition, this study’s results portray the psychological toll arising from the pain and difficulties caused by the treatment and isolation from daily life during the treatment period. These findings show that emotional and psychological nursing needs were most frequently met for parents in the initiation of treatment and post-treatment phases. Hence, nursing care is highly recommended for resolving psychosocial problems and adaptation after treatment as well [31].

It was found that parents of pediatric cancer patients had different nursing needs depending on the treatment phase, which implies that tailored education for parents and guardians by treatment phase is necessary as opposed to a one-time explanation at the time of diagnosis. The continuity of education should also be ensured, and the counseling system should provide caregivers with diverse plans according to the patients’ activities of daily living. As the frequency of hospital visits decreases post-treatment and parents have fewer opportunities to meet medical staff, education using the internet is suggested beyond the on-site cancer education center in hospitals. Since patients share limited information about themselves using social networking services [32], it is highly suggested to provide a nursing education platform that systematically utilizes social networking technology.

## 5. Conclusions

This study applied Mishel’s theory of uncertainty to parents of childhood cancer patients and investigated differences in uncertainty levels across treatment phases in 119 subjects. A significant difference was found in the uncertainty levels of parents of pediatric cancer patients according to the treatment phase. The empirical findings suggest the need to develop tailored education for caregivers to reduce the uncertainty of parents of pediatric cancer patients during each treatment phase.

While most studies have investigated nursing needs by dividing them into therapeutic and emotional domains, this study explored parents’ practical needs using open-ended questions. This approach was useful in expanding our understanding of the nursing needs of parents of pediatric cancer patients. It was found that the highest-priority nursing needs were related to parents’ and patients’ daily lives, such as protocols for infection control in their home and diet. Therefore, a definite need exists for serial, systematic education that meets parents’ needs during each treatment phase.

## Figures and Tables

**Table 1:** Demographic characteristics of the participants (*n* = 119).

Characteristics	Categories	*n* (%)	Median/Mean ± SD
Relationship with the pediatric cancer patient	Father	14 (11.8)	
	Mother	105 (88.2)	
Age (years)	20–29	4 (3.4)	39/39.36 ± 5.42
	30–39	56 (47.1)	
	40–49	56 (47.1)	
	≥50	3 (2.5)	
Religious	None	58 (48.7)	
	Yes	61 (51.3)	
Education	≤Middle school	2 (1.7)	
	High school	23 (19.3)	
	≥College/University	94 (79.0)	
Occupation	Employed	35 (29.4)	
	Unemployed	84 (70.6)	
Average monthly household income (KRW 10,000)	≤199	21 (17.6)	
	200–299	21 (17.6)	
	300–399	27 (22.7)	
	≥400	50 (42.0)	

**Table 2:** Characteristics of Children with Pediatric Cancer (*n* = 119).

Characteristics	Categories	*n* (%)	Median/Mean ± SD
Gender	Male	69 (58.0)	
	Female	50 (42.0)	
Type of cancer	Acute lymphoblastic leukemia	29 (24.4)	
	Acute myeloid leukemia	13 (10.9)	
	Acute biphenotypic leukemia	4 (3.4)	
	Chronic myeloid leukemia	1 (0.8)	
	Brain tumor	16 (13.4)	
	Non-Hodgkin lymphoma	14 (11.8)	
	Osteosarcoma	8 (6.7)	
	Neuroblastoma	8 (6.7)	
	Retinoblastoma	7 (5.9)	
	Rhabdomyosarcoma	6 (5.0)	
	Germ cell tumor	5 (4.2)	
	Wilms tumor	4 (3.4)	
	Ewing sarcoma	3 (2.5)	
	Hepatoblastoma	1 (0.8)	
Age	0–11 months	7 (5.9)	3/7.65 ± 4.98
	1–5 years	36 (30.3)	
	6–10 years	33 (27.7)	
	≥11 years	43 (36.1)	
Order of birth	First	73 (61.3)	
	Second	36 (30.3)	
	≥Third	10 (8.4)	
Time since diagnosis	1–11 months	65 (54.6)	
	≥12 months	54 (45.4)	
Relapse	Yes	23 (19.3)	
	No	96 (80.7)	
Transplantation	Yes	35 (29.4)	
	No	84 (70.6)	

**Table 3:** Uncertainty score according to treatment phases in each group (*n* = 119).

Subdomain	Group 1 ^a^ (*n* = 39)	Group 2 ^b^ (*n* = 40)	Group 3 ^c^ (*n* = 40)	F	*p* (*Post-Hoc* Test: Scheffé)
	M ± SD				
Ambiguity	37.29 ± 8.04	36.80 ± 8.34	31.60 ± 6.85	7.49	0.001 (a, b > c)
Lack of clarity	24.28 ± 4.21	23.43 ± 4.47	21.75 ± 4.48	3.41	0.036 (a > c)
Lack of information	11.87 ± 2.71	10.23 ± 2.35	10.48 ± 2.35	5.06	0.008 (a > b, c)
Unpredictability	13.69 ± 2.70	12.88 ± 3.23	11.53 ± 2.49	5.94	0.003 (a > c)
Total	87.77 ± 13.43	83.33 ± 15.10	75.35 ± 12.82	8.20	<0.001 (a, b > c)

^a^ Group 1 = treatment initiation phase; ^b^ Group 2 = ongoing treatment phase; ^c^ Group 3 = post-treatment phase

**Table 4:** Scores for each item on PPKS-FM scale across the treatment phase.

Item		Group 1 (*n* = 39) ^a^	Group 2 (*n* = 40) ^b^	Group 3 (*n* = 40) ^c^
		Mean ± SD		
**Ambiguity**: 13 questions				
1.	I am unsure if my child’s illness is getting better or worse	3.44 ± 1.23	3.40 ± 1.11	2.78 ± 1.14
2.	It is unclear how bad my child’s pain will be	3.72 ± 1.19	3.63 ± 1.03	2.68 ± 1.16
3.	My child’s symptoms continue to change unpredictably	2.64 ± 1.11	2.73 ± 1.04	2.08 ± 0.97
4.	It is difficult to know if the treatments or medications my child is getting are working	2.64 ± 0.96	2.65 ± 1.10	2.60 ± 0.98
5.	Because of the unpredictability of my child’s illness, I cannot plan for the future	2.90 ± 1.19	2.88 ± 1.02	2.75 ± 1.10
6.	The course of my child’s illness keeps changing. He/she has good and bad days	2.72 ± 1.05	2.83 ± 1.06	2.30 ± 0.99
7.	It’s vague to me how I will manage the care of my child after he/she leaves the hospital	2.97 ± 1.22	2.33 ± 1.02	2.25 ± 0.98
8.	It is not clear what is going to happen to my child	3.46 ± 1.00	3.28 ± 1.04	2.93 ± 1.10
9.	The results of my child’s tests are inconsistent	2.69 ± 1.13	2.38 ± 0.90	2.03 ± 0.83
10.	The effectiveness of the treatment is undetermined	2.36 ± 0.81	2.40 ± 1.01	1.80 ± 0.61
11.	It is difficult to determine how long it will be before I can care for my child by myself	2.90 ± 0.97	2.48 ± 1.13	1.83 ± 0.81
12.	Because of the treatment, what my child can and cannot do keeps changing	3.46 ± 1.14	3.88 ± 0.72	3.68 ± 1.02
13.	I’m certain they will not find anything else wrong with my child	2.03 ± 1.18	1.98 ± 1.07	1.93 ± 0.92
**Lack of clarity**: nine questions				
14.	I have a lot of questions without answers	4.21 ± 1.00	3.85 ± 0.83	3.73 ± 0.96
15.	The explanations they give about my child seem hazy to me	2.36 ± 0.96	2.63 ± 0.95	2.23 ± 0.86
16.	The purpose of each treatment for my child is clear to me	2.51 ± 0.68	2.53 ± 0.68	2.20 ± 0.76
17.	I do not know when to expect things will be done to my child	3.54 ± 1.02	3.45 ± 0.93	2.63 ± 1.03
18.	I understand everything explained to me	2.59 ± 0.72	2.43 ± 0.75	2.38 ± 0.81
19.	The doctors say things to me that have many meanings	2.33 ± 0.81	2.40 ± 0.84	2.40 ± 1.00
20.	There are so many different types of staff; it’s unclear who is responsible for what	2.23 ± 0.84	2.15 ± 0.98	2.18 ± 0.90
21.	I can depend on the nurses when I need them	2.13 ± 1.06	1.95 ± 0.75	1.95 ± 0.68
22.	The doctors and nurses use everyday language so I can understand what they are saying	2.38 ± 0.78	2.05 ± 0.64	2.08 ± 0.69
**Lack of information**: five questions				
23.	I don’t know what is wrong with my child	2.08 ± 1.13	1.93 ± 0.80	2.05 ± 1.01
24.	My child’s treatment is too complex to figure out	2.82 ± 0.79	2.35 ± 0.98	2.10 ± 0.74
25.	They have not given my child a specific diagnosis	2.44 ± 1.19	2.10 ± 1.01	2.08 ± 1.05
26.	My child’s diagnosis is definite and will not change	2.49 ± 0.94	2.13 ± 0.94	2.35 ± 1.10
27.	The seriousness of my child’s illness has not been determined	2.05 ± 1.03	1.73 ± 0.75	1.90 ± 0.81
**Unpredictability**: four questions				
28.	I can predict how long my child’s illness will last and feel ill	3.69 ± 0.86	3.38 ± 1.17	3.40 ± 1.01
29.	I usually don’t know if my child is going to have a good or bad day	3.33 ± 1.13	3.30 ± 1.11	2.78 ± 1.07
30.	I can generally predict the course of my child’s illness	3.18 ± 1.00	3.03 ± 1.05	2.73 ± 0.91
31.	My child’s physical distress is unpredictable; I don’t know when it is going to get better or worse	3.49 ± 0.97	3.18 ± 0.87	2.63 ± 1.03

^a^ Group 1 = treatment initiation phase; ^b^ Group 2 = ongoing treatment phase; ^c^ Group 3 = post-treatment phase.

**Table 5:** Uncertainty-related themes described by participants according to the treatment phase.

Treatment Phase	Theme (Frequency)	Nursing Needs
Total	Diet for infection control (68)	Availability of the same meal as usual
		Low-bacterial diet during the nontreatment period
	Environmental management and activity restrictions to prevent infection (65)	Specific methods of environmental management
		The range of normal activity in daily life after discharge
	Current health condition and prognosis (52)	Specific information on the patient’s current condition and prognosis
		Possibility of recurrence
Treatment initiation group	Prognosis and possibility of adoption to normal life (19)	Possibility to return to ordinary life as before
		Probability of a full recovery after treatment completion
	Infection prevention rules for daily activities (14)	Infection control methods at home (cleaning method, bedding management)
		Effective methods for environmental management and infection prevention after discharge
Ongoing treatment group	Progress of treatment and medication (34)	The severity of the disease currently (compared to the time of diagnosis)
		Curiosity about the reasons for and side effects of a new procedure or medication
	Caring for your child’s psychological state (14)	Child’s mental health and psychological care
		How to play with a child with illness: emotional support is needed
	Adverse effects of treatment and coping strategy (9)	Ways of alleviating the side effects of treatment
Post-treatment group	Prognosis after termination and re-adaptation to school or society (19)	How a child receives care after treatment and how much and how much
		Degree of risk of recurrence
		Allowable activity in daily life after treatment is over
	Long-term management method and system for disease-related sequelae (15)	Description of post-treatment period from a long-term perspective
		Availability of detailed explanations and important points through education, lectures, and social media
		Complication relief program
	Emotional support after treatment ends (10)	Psychotherapy for social adaptation
		Relieving a child of loneliness and depression

The numbers in parentheses e.g., “(7)” are the number of times the respondent describes.

## Data Availability

Data are available upon request.
